# Transcriptomic analysis of methyl jasmonate treatment reveals gene networks involved in drought tolerance in pearl millet

**DOI:** 10.1038/s41598-022-09152-6

**Published:** 2022-03-25

**Authors:** Adama Ndiaye, Amadou Oury Diallo, Ndèye Coura Fall, Rose Diambogne Diouf, Diaga Diouf, Ndjido Ardo Kane

**Affiliations:** 1 Centre d’Étude Régional Pour L’Amélioration de L’Adaptation À La Sècheresse (CERAAS), Institut Sénégalais de Recherches Agricoles (ISRA), Route de Khombole, Thiès, BP 3320 Sénégal; 2 Laboratoire Campus de Biotechnologies Végétales, Département de Biologie Végétale, Faculté Des Sciences Et Techniques, Université Cheikh Anta Diop (UCAD), 10700 Dakar-Fann, Dakar, Sénégal; 3 Laboratoire Mixte International Adaptation Des Plantes Et Des Microorganismes Associés Aux Stress Environnementaux (LAPSE), Dakar, Sénégal

**Keywords:** Molecular biology, Plant sciences

## Abstract

Water deficit stress at the early stage of development is one of the main factors limiting pearl millet production. One practice to counteract this limitation would be to resort to the application of hormones to stimulate plant growth and development at critical stages. Exogenous methyl jasmonate (MeJA) can improve drought tolerance by modulating signaling, metabolism, and photosynthesis pathways, therefore, we assumed that can occur in pearl millet during the early stage of development. To decipher the molecular mechanisms controlling these pathways, RNAseq was conducted in two pearl millet genotypes, drought-sensitive SosatC88 and drought-tolerant Souna3, in response to 200 μM of MeJA. Pairwise comparison between the MeJA-treated and non-treated plants revealed 3270 differentially expressed genes (DEGs) among 20,783 transcripts in SosatC88 and 127 DEGs out of 20,496 transcripts in Souna3. Gene ontology (GO) classification assigned most regulated DEGs in SosatC88 to heme binding, oxidation–reduction process, response to oxidative stress and membrane, and in Souna3 to terpene synthase activity, lyase activity, magnesium ion binding, and thylakoid. The Kyoto Encyclopedia of Genes and Genomes (KEGG) enrichment analysis reveals that DEGs in SosatC88 are related to the oxidation–reduction process, the biosynthesis of other secondary metabolites, the signal transduction, and the metabolism of terpenoids, while in Souna3, DEGs are related to the metabolism of terpenoids and the energy metabolism. Two genes encoding a diterpenoid biosynthesis-related (Pgl_GLEAN_10009413) and a Glutathione S transferase T3 (Pgl_GLEAN_10034098) were contra-regulated between SosatC88 and Souna3. Additionally, five random genes differentially expressed by RNAseq were validated using qPCR, therefore, they are potential targets for the development of novel strategies breeding schemes for plant growth under water deficit stress. These insights into the molecular mechanisms of pearl millet genotype tolerance at the early stage of development contribute to the understanding of the role of hormones in adaptation to drought-prone environments.

## Introduction

Worldwide, water deficit contributes to crop production losses, particularly in the Sub-Saharan Africa countries, where agriculture depends on rainfall. In addition, population growth increases food demand while arable land is continuously shrinking. Meanwhile, climate forecasts are predicted to be extremes by 2050^1^ in Africa, therefore, there is a need to breed high-yielding drought-tolerant genotypes. One of the major staple food and drought-tolerant plant is pearl millet (*Pennisetum glaucum* L. Br), ranked fourth among the cultivated tropical cereals. Since its domestication from West Africa 4,900 years ago^2^, pearl millet constitutes a staple food for more than 100 million people, with 90% grown by smallholder farmers^3^, hence contributing to nutrition and food security. Indeed, its grain is gluten-free, rich in vitamins, micronutrients (iron and zinc), and protein (8–19%)^4^. The genome size of the crop is estimated to 1.79 gigabytes containing 38,579 genes and recent studies led to the identification of specific marker-traits linked to key agronomic traits, such as grain yield, stem and leaf biomass as well as tremendous adaptive traits for heat and drought^5^. As a cross-pollinated C4 plant (2n = 2x = 14), pearl millet endows with a large genetic diversity and grows in some of the hottest and driest areas across the arid and semi-arid regions. Its vulnerability to drought is genotype dependent^6^.

Drought-related stresses at early stage of pearl millet plant development are limiting factors to its production^7^. Water deficit for example induces a limitation of photosynthesis activities due to reduced leaf expansion, impaired photosynthetic machinery, premature leaf senescence^8,9^, stomatal closure^10^, and the activity of Calvin’s cycle enzymes such as Rubisco and phosphoenolpyruvate carboxylase^11^. Reactive oxygen species (ROS), produced under water deficit, target various organelles, including chloroplasts, mitochondria, and peroxisomes, resulting in cell membrane instability, senescence, or plant death^12^. Other physiological responses to water deficit include decreasing stem height and diameter, leaf number, leaf area ratio, dry matter, shoot/root weight ratio, net CO_2_ assimilation and chlorophyll fluorescence, photosynthetic activity, altered cell wall elasticity and generation of toxic metabolites^13,14^. Another important factor leading to plant tolerance to water deficit is hormonal regulation. The expression of several transcription factors and their target genes involved in mediating phytohormones abscisic acid (ABA) under water deficit conditions, key components of perception and signaling, are induced to attenuate the negative impacts due to water deficit^13,15^.

Exogenous treatment of abscisic acid^16^ and salicylic acid^17^ improves tolerance under water deficit in wheat (*Triticum aestivum*). The application of jasmonates induced improved drought tolerance in barley (*Hordeum vulgare*),^18^, soybean (*Glycine max*)^19^, and sugar beet (*Beta vulgaris*)^20^. A treatment of Methyl jasmonate (MeJA) can enhance tolerance to dehydration of plants subjected to osmotic stress caused by polyethylene glycol^21^ and water retention^22^. MeJA governs many aspects of plant development, including seed germination, root growth, flowering, fruit ripening, senescence^23,24^ and abiotic stresses tolerance^25^. This hormone mitigates the influence of water deficit by promoting stomatal closure induced by H_2_O_2_ generation^26^, acting principally on the expression of genes involved in signaling processes.

Transcriptomic studies in pearl millet have been used to reveal responsive genes under drought, heat, or salinity conditions^27,28^. However, the mechanism of pearl millet in response to MeJA treatment has a negligible focus. Proteome has also been analyzed in pearl millet that provided insights into the functional enzyme involves in drought responses. Comprehensive physiological and proteomic responses of the drought stressed pearl millet revealed significant tissue-specific signatures during the adaptation to drought conditions^29^. In addition, a stay-green phenotype signature in drought-tolerant pearl millet was defined that contributes to a significant effect on yield, grain filling and chlorophyll loss^30^. The present study investigates genes that are differentially expressed after an exogenous MeJA treatment and with a mimic of water deficit by withholding water supply to decipher the molecular basis modulating the response of pearl millet under water scarcity conditions. By spraying pearl millet plants at the early stage of development with MeJA, the assumption that a differential gene expression could activate or repress genes underlying key metabolic and biosynthesis pathways that lead to coping with water deficit-related stress was tested. Comparative transcriptome analysis of two pearl millet genotypes contrasting by their level of tolerance to water deficit at the early stage identified sets of differentially expressed genes (DEGs) between non-treated and MeJA-treated plants. The regulation of DEGs modulated by MeJA and new genetic insights of adaptive mechanisms of pearl millet in response to water deficit stress are discussed.

## Results

### MeJA effects at early-stage development of pearl millet

**Figure 1:**
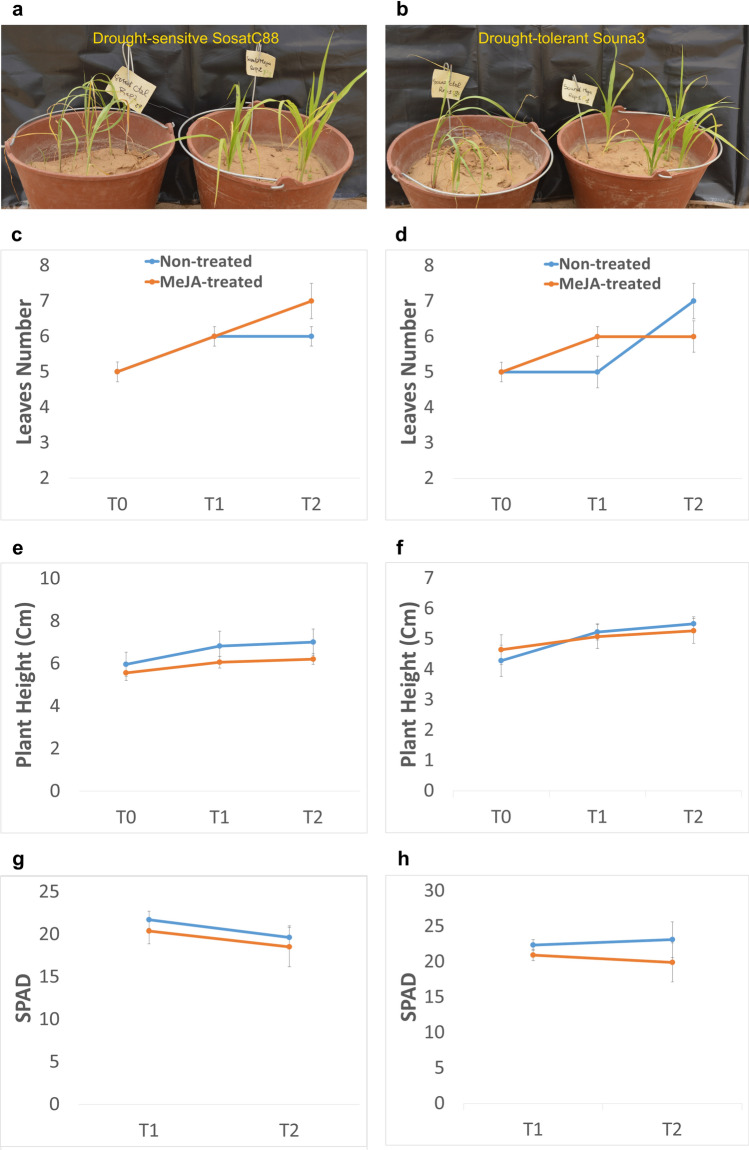
Effects of MeJA spraying on development and chlorophyll content of SosatC88 (left: non-treated; right: MeJA-treated) and Souna3 (left: non-treated; right: MeJA-treated).

Plants of two genotypes contrasting for the response to drought (sensitive SosatC88 and tolerant Souna3) showed no significant developmental changes for leaves number and plant height, p > 0.05 at 17 days after sowing, i.e. T0. The aerial part of these plants was then daily sprayed with MeJA (200 µM) for 10 days over T0 and we observed that the non-treated plants of both SosatC88 and Souna3 appeared withered compared to those treated (Fig. 1a,b). Plant height, leaves number and chlorophyll content were measured in both genotypes. MeJA did not significantly change leaves number in SosatC88 at T1, i.e. five days after treatment (p = 0.72), and T2, i.e. 10 days after treatment (p = 0.052). However, in Souna3 at T1 MeJA-treated plants have significantly more leaves (p = 0.049) than non-treated plants (Fig. 1c,d). In both genotypes, the height of MeJA-treated plants stayed lower than that of non-treated plants until the end of the treatment with a significant difference observed (*p* = 0.03) in SosatC88 at T2 (Fig. 1e,f) in treated and non-treated plants. In both genotypes, the chlorophyll content decreased during the 10 days of water deficit and remained lower in MeJA-treated plants, however, this change was only significant in Souna3 at T1 (*p* = 0.03) (Fig. 1g,h).

### MeJA treatment in modulating gene expression

To evaluate whether the MeJA treatment in both SosatC88 and Souna3 mimicked drought tolerance response at the gene expression level, we conducted qPCR using RNA extracted from the aerial part of plants sprayed or not for 10 days with MeJA. The relative expression levels of pearl millet lipoxygenase 2 (*PgLox2*) and of 9-cis-epoxycarotenoid (*PgNCED*) were determined. The two genes were previously used as endogenously regulated after a MeJA treatment or in response to drought-induced stress, respectively. The results showed that *PgLox2* was over-expressed in MeJA-treated plants compared to the non-treated (Supplemental figures, Fig. S1), indicating that MeJA penetrated pearl millet plants and did change gene expression. A significant positive fold change *PgNCED* expression profile was observed in both drought-sensitive SosatC88 and drought-tolerant Souna3, indicating that the MeJA treatment modules the response to drought-induced stress in pearl millet by modulating gene expression (Supplemental figures, Fig. S1).

### RNA-seq assembly and analysis after MeJA treatment

To further explore this contrasting behavior between genotypes, we analyzed the transcriptomic responses to MeJA treatment on drought-sensitive SosatC88 and drought-tolerant Souna3. A total of 408,935.888 raw reads were generated, with an average of 34,077.991 reads per sample. After filtration and quality check, 379,364.208 high-quality reads were obtained with an average of 30,624.486 reads per sample. On average, 92.77% of total data passed a Phred score of ≥ Q30 bases for each sample. A final number of 367,493.828 high-quality reads (passing filter and correctly assigned to a sample after demultiplexing) were then aligned onto the pearl millet reference genome and 89.29–90.81% reads from twelve samples were uniquely mapped (Supplemental file, Table S1).

**Figure 2:**
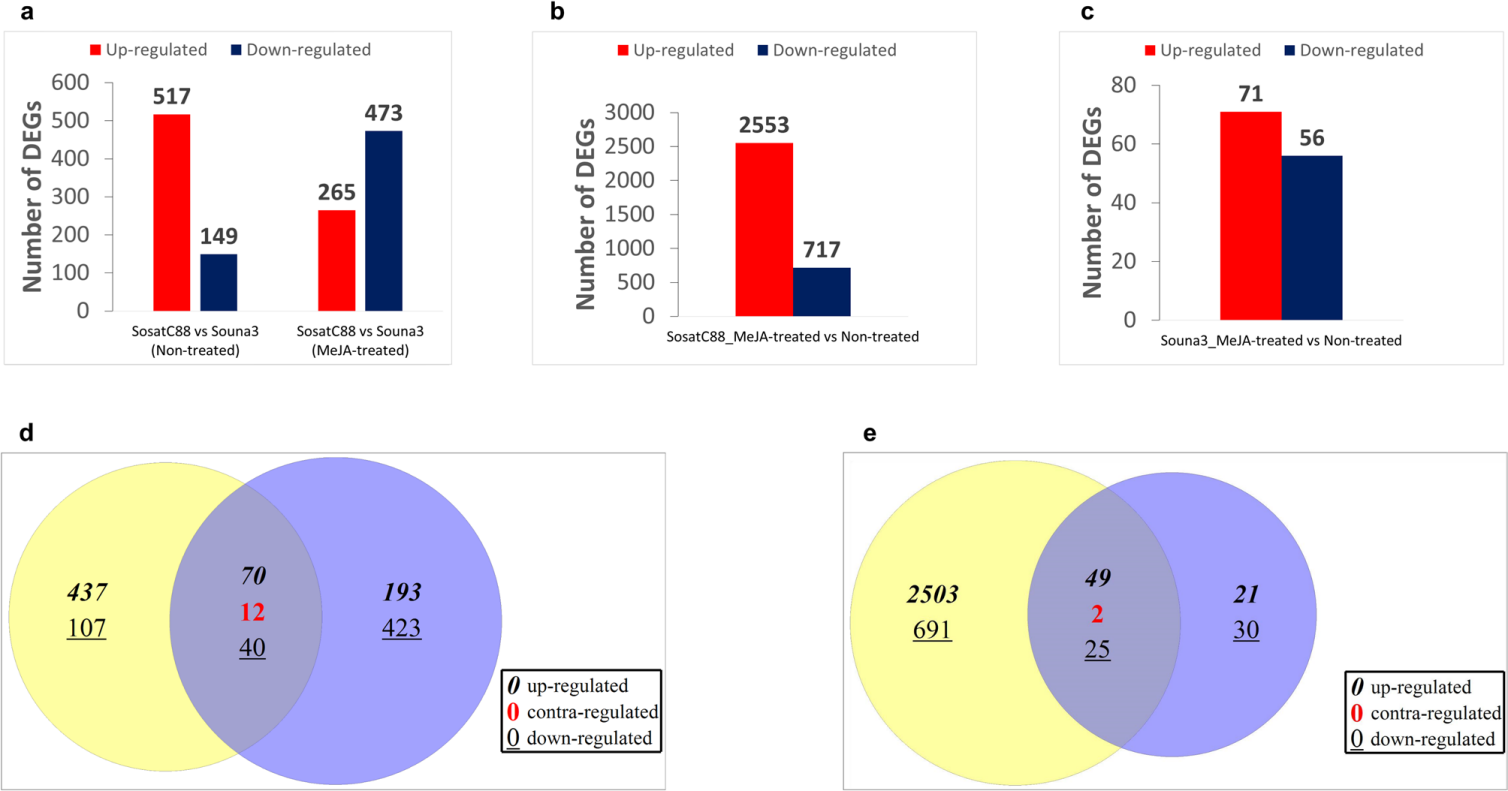
(**a**, **b** and **c**) Number of DEGs in all combinations with fold change > 2 or < -2 and FDR-corrected *p*-value < 0.05) Red and blue bars indicate up-regulated and down-regulated respectively; (**d**) Venn diagram of number of differentially expressed transcripts in ‟SosatC88 vs Souna3 (non-treated)” (yellow) and ‟SosatC88 vs Souna3 (MeJA-treated)” ( blue); (**e**) Venn diagram of number of differentially expressed transcripts in ‟SosatC88_MeJA- treated vs non-treated” ( yellow) and ‟Souna3_MeJA- treated vs non-treated” (blue).

Differentially expressed genes (DEGs) for all samples were identified using edgeR package with |log2FoldChange|> 2 and p-adj < 0.05. In the drought-sensitive SosatC88, 3270 of 20,783 transcripts were differentially expressed (717 down-regulated and 2553 up-regulated). In drought-tolerant Souna3, 127 of 20,496 transcripts were differentially expressed (56 down-regulated and 71 up-regulated). This indicates that there were more differential genes in the drought-sensitive SosatC88 than in the drought-tolerant Souna3. Pairwise comparison (Fig. 2a-c) between non-treated SosatC88 and Souna3 plants revealed a total of 517 up-regulated genes and 149 down-regulated genes. Between MeJA-treated SosatC88 and Souna3*,* 265 genes are up-regulated and 473 genes are down-regulated. Comparison from non-treated SosatC88 and Souna3 plants and MeJA-treated SosatC88 and Souna3 revealed that 70 and 40 genes are up-regulated and down-regulated, respectively. However, 12 genes were contra-regulated (Fig. 2d, Supplemental figures). Comparison from SosatC88 and Souna3 DEGs showed 49 and 25 genes are up-regulated and down-regulated, respectively. However, two genes encoding a diterpenoid biosynthesis-related (Pgl_GLEAN_10009413) and a Glutathione S transferase T3 (Pgl_GLEAN_10034098), respectively, were contra-regulated between SosatC88 and Souna3 (Fig. 2e, Supplemental figures).

### DEGs functional analysis after MeJA treatment

**Figure 3:**
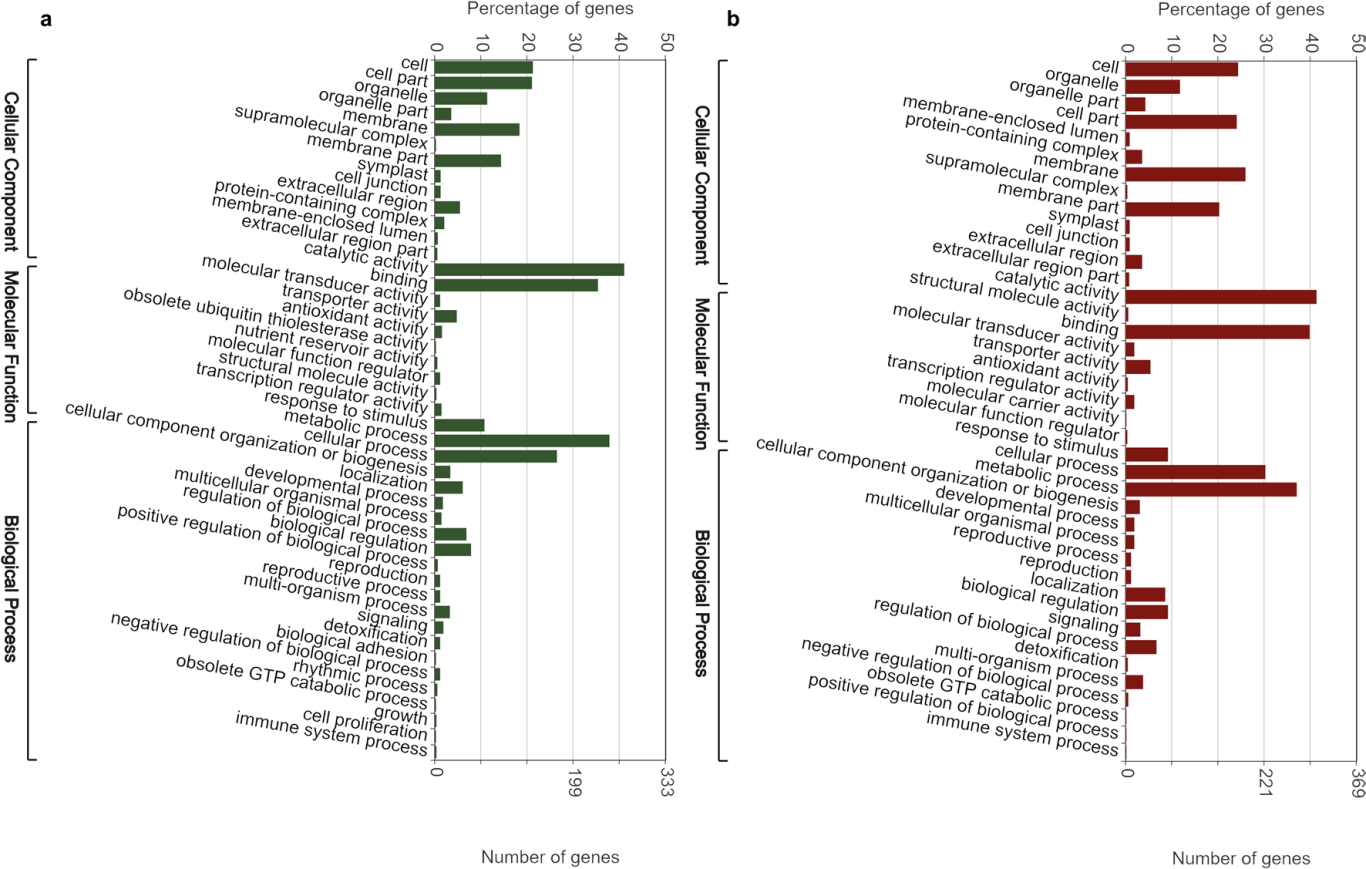
Gene ontology distribution of DEGs: (**a**) SosatC88vs Souna3 (non-treated); (**b**) SosatC88 vs Souna3 (MeJA-treated).

**Figure 4:**
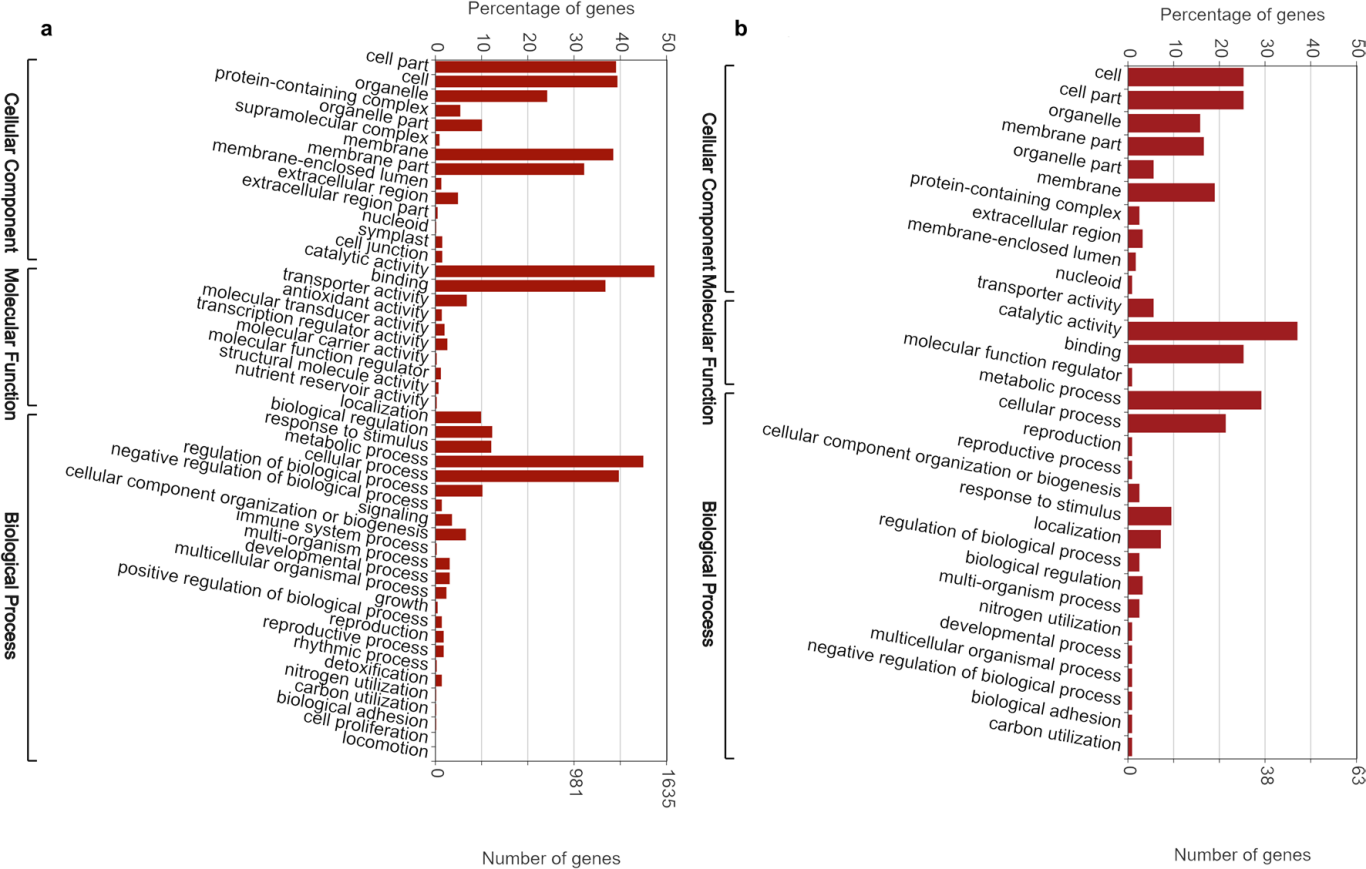
Gene ontology distribution of DEGs: (**a**) SosatC88_MeJA-treated vs non-treated; (**b**) Souna3_MeJA-treated vs non-treated.

Gene ontology (GO) assignments of biological process (BP), cellular component (CC) and molecular function (MF) were used to classify the differentially expressed genes and their expected functions from the datasets identified above. GO classification of pearl millet gene lists from DEGs were also carried out. Comparing non-treated SosatC88 and non-treated Souna3 , a total of 45 GO terms was assigned (13 CC, 10 MF and 22 BP) (Fig. 3a), 40 GO terms between MeJA-treated SosatC88 and MeJA-treated Souna3 (13 CC, 9 MF and 18 BP) (Fig. 3b), 48 GO terms between non-treated and MeJA-treated SosatC88(14 CC, 10 MF and 24 BP) (Fig. 4a), and 30 GO terms between non-treated and MeJA-treated Souna3 (10 CC, 4 MF and 16 BP) (Fig. 4b). This classification reveals that in non-treated conditions, differences between the two genotypes are particularly in cell (141 genes) cell part (140 genes), membrane (122 genes), catalytic activity (273 genes), binding (235 genes), metabolic process (252 genes) and cellular process (176 genes). In MeJA-treated conditions, the most differences appeared in cell (179 genes), cell part (177 genes), membrane (191 genes), membrane part (149 genes), catalytic activity (305 genes), binding (294 genes), cellular process (223 genes), and metabolic process (273 genes) indicating that some important cellular processes and metabolic activities occurred in the leaves of water-stressed millet in response to MeJA treatment.

According to the GO ontology, MeJA treatment modulated several genes involved in cell part, cell, organelle, protein-containing complex, organelle part, membrane, membrane part, extracellular region, catalytic activity, binding, transporter activity, localization, biological regulation, response to stimulus, metabolic process, cellular process, regulation of the biological process, signaling, cellular component organization or biogenesis. In drought-sensitive SosatC88, the DEGs are greater than in drought-tolerant Souna3 (Fig. 4a,b). However, MeJA treatment during non-watered conditions has a particular impact on the genes involved in the structure of the membrane and the binding genes.

### GO terms enrichment analysis

**Figure 5:**
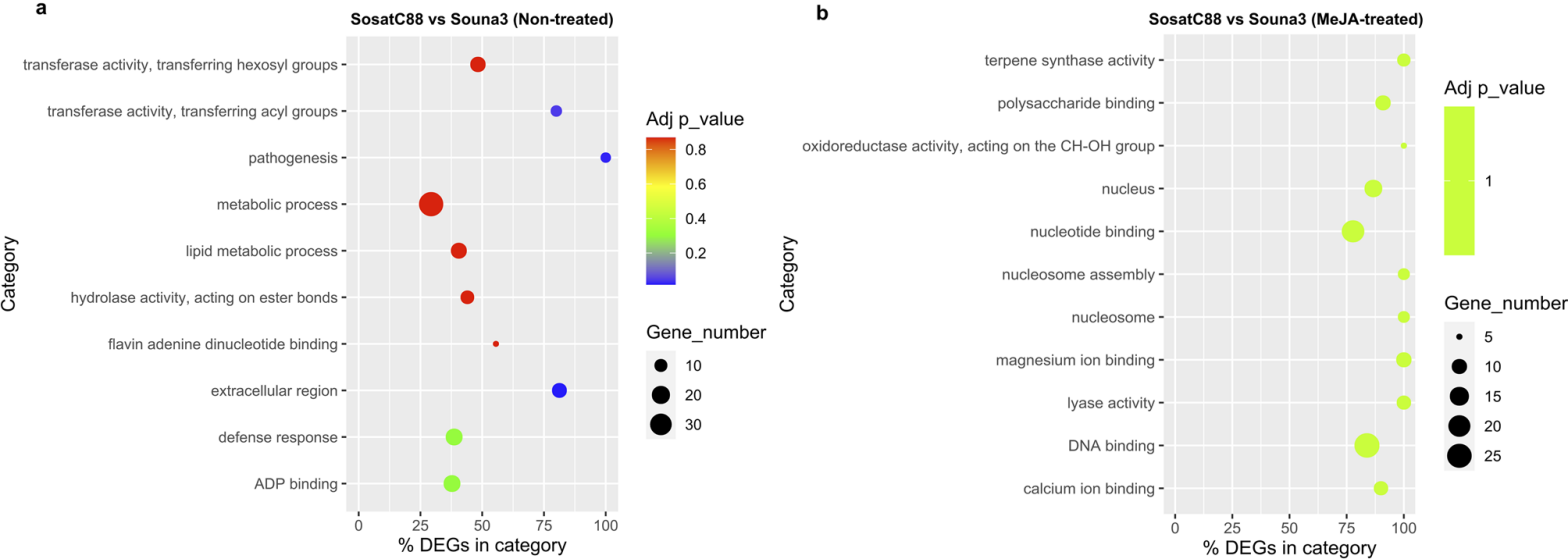
(**a**) The top 10 over-represented GO terms under non-treated condition between SosatC88 and Souna3. (**b**) The top 10 over-represented GO terms under MeJA-treated condition between SosatC88 and Souna3. The Adj p-value is the corrected p-value ranging from 0 to 1.

Goseq analysis was used to classify the functions of DEGs. Between both non-treated SosatC88 and Souna3, with related terms ‛extracellular region’ (cellular component category), ‛pathogenesis’ (biological process category) and ‛transferase activity, transferring acyl group’ (molecular function category) were significantly enriched (Fig. 5a). Between MeJA-treated SosatC88 and Souna3, none of the categories are significantly enriched; but molecular function category including ‛magnesium ion binding’, ‛lysase activity’, ‛terpene synthase activity’, ‛DNA binding’, ‛nucleotide binding’, ‛polysaccharide binding’, ‛calcium binding’, ‛oxidoreductase activity, acting on the CH-OH group of donors, NAD or NADP as acceptor’ and cellular component including ‛nucleus’ and ‛nucleosome’ were the top over-represented categories (Fig. 5b).

**Figure 6:**
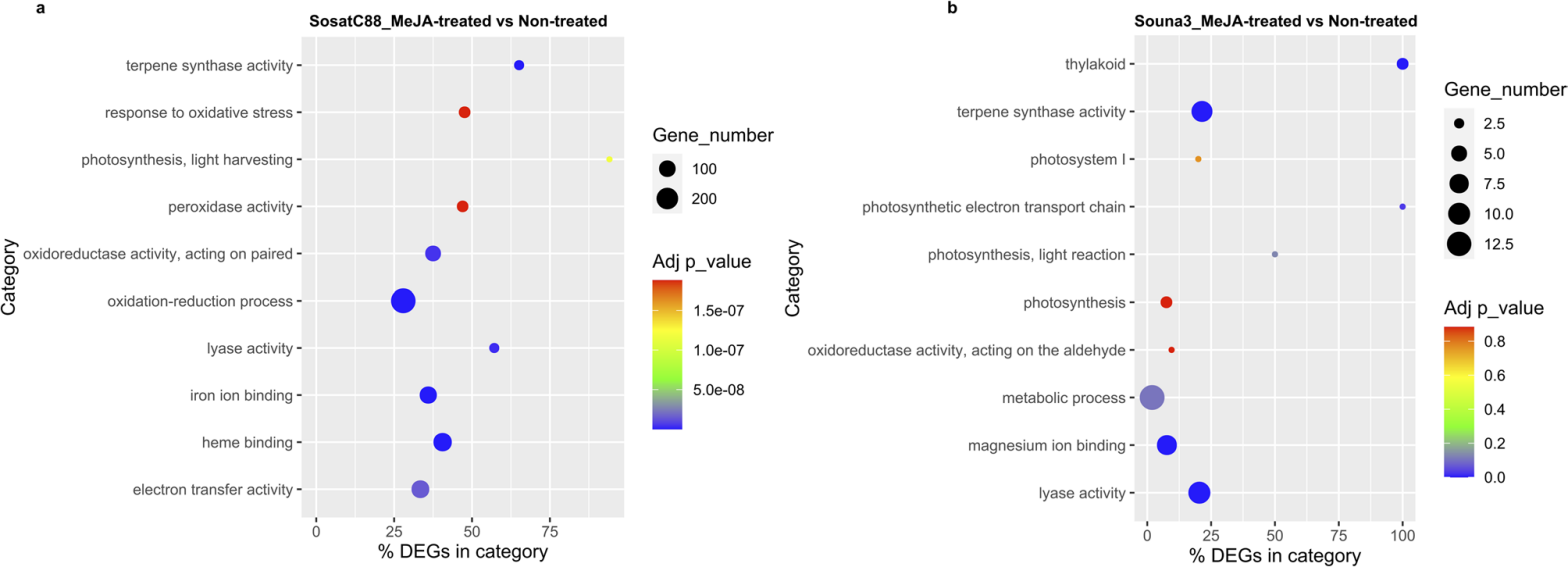
(**a**) The top 10 over-represented GO terms in SosatC88_MeJA-treated-vs-non-treated. (**b**) The top 10 over-represented GO terms in Souna3_MeJA-treated-vs-non-treated. The Adj p-value is the corrected p-value ranging from 0 to 1.

Between SosatC88 MeJA-treated and non-treated, molecular function category including ‛heme binding’, ‛iron ion binding’, ‛terpene synthase activity’, ‛lyase activity’ ‛oxidoreductase activity, acting on paired donors, with incorporation or reduction of molecular oxygen’, ‛electron transfer activity’, ‛peroxidase activity’ and biological process category including ‛oxidation–reduction process’, ‛photosynthesis, light harvesting’, ‛response to oxidative stress’ were the most highly enriched (Fig. 6a). On the other hand, molecular function category, ‛terpene synthase activity’, ‛lyase activity’, ‛magnesium ion binding’; cellular component category, ‛thylakoid’ and biological process ‛photosynthetic electron transport chain’ were significantly enriched in Souna3_MeJA-treated vs non-treated. The GO molecular function category, ‛terpene synthetase activity’ and ‛lysase activity’ were also highly enriched in both SosatC88_MeJA-treated vs non-treated and Souna3_MeJA-treated vs non-treated (Fig. 6b).

### Pathways analysis

**Figure 7:**
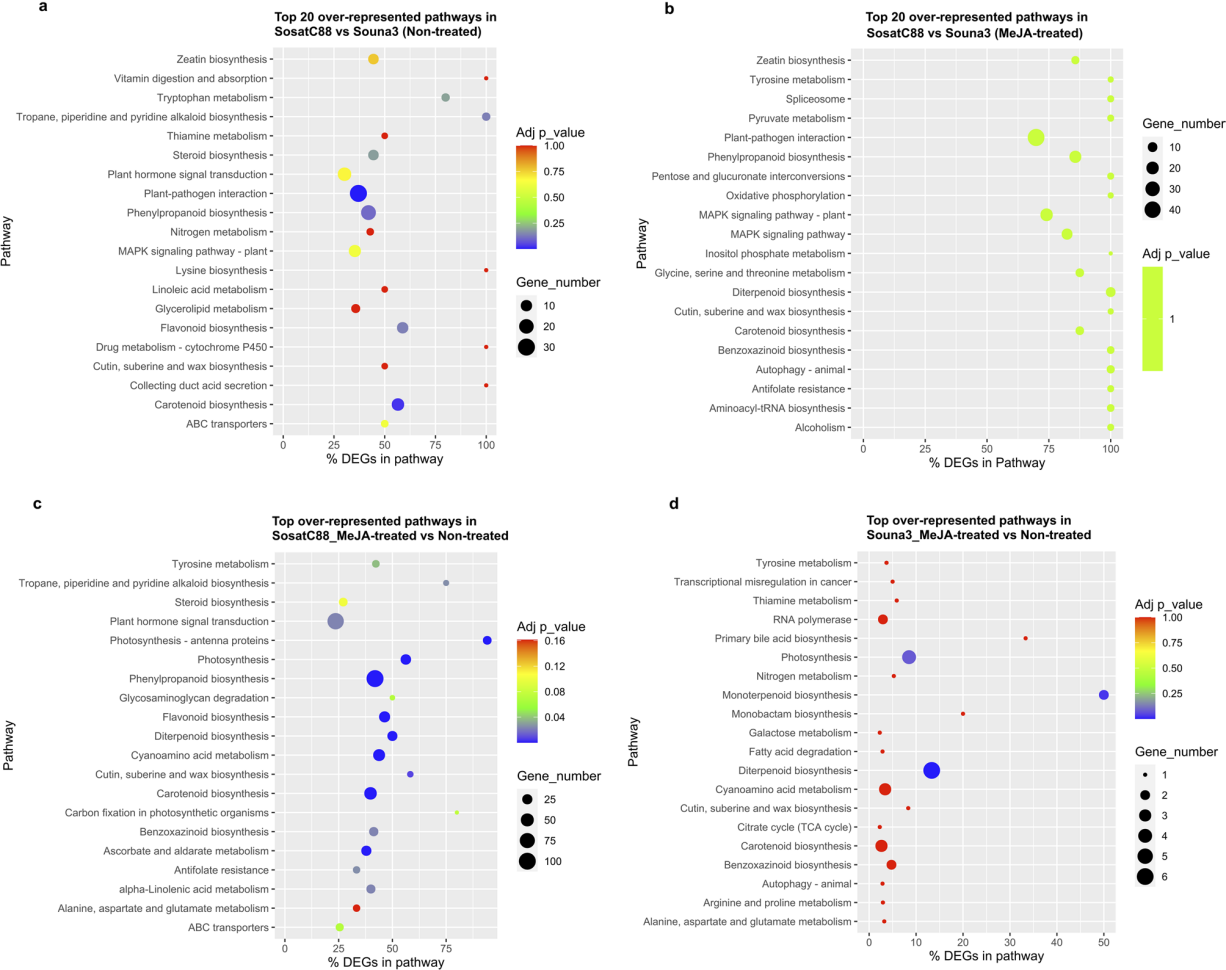
KEGG pathway enrichment analysis of the differentially expressed genes (DEGs)^72^.

Based on the KEGG database, several signaling pathways and genes involved in the response of both millet varieties to MeJA treatment in water-stressed conditions were discovered. Comparing non-treated SosatC88 and Souna3 and MeJA-treated SosatC88 and Souna3, an increase in carbon metabolism, pyruvate metabolism, oxidative phosphorylation was observed (Fig. 7a,b and Supplementary file). Propanoate metabolism, inositol phosphate metabolism, carbon fixation in photosynthetic organisms, biosynthesis of unsaturated fatty acids are induced by MeJA treatment. The most representative pathways of the overexpressed genes in between non-treated and MeJA-treated SosatC88 and non-treated and MeJA-treated Souna3 were those associated with metabolism (KO 01100), biosynthesis of secondary metabolites (KO 01110), antibiotic biosynthesis (KO 01,130), carbon metabolism (KO 01,200), photosynthesis (KO 00,195) and plant hormone signal transduction (KO 04,075) (Fig. 7c,d and Supplementary file). Some enriched pathways such as oxidative phosphorylation (KO 00,190), carbon fixation in photosynthetic organisms (KO 00,710), phenylalanine metabolism (KO 00,360), phenylalanine tyrosine tryptophan metabolism (KO 00,400), phenylpropanoid (KO 00,940), ubiquitin-mediated proteolysis (KO 04,120), MAPK signaling (KO 04,016) and circadian rhythm (KO 04,712) are only represented in SosatC88, indicating that these pathways were recruited by the MeJA hormone to enhance SosatC88 adaptively under water deficit.

### Validation of gene expression using qPCR

**Figure 8:**
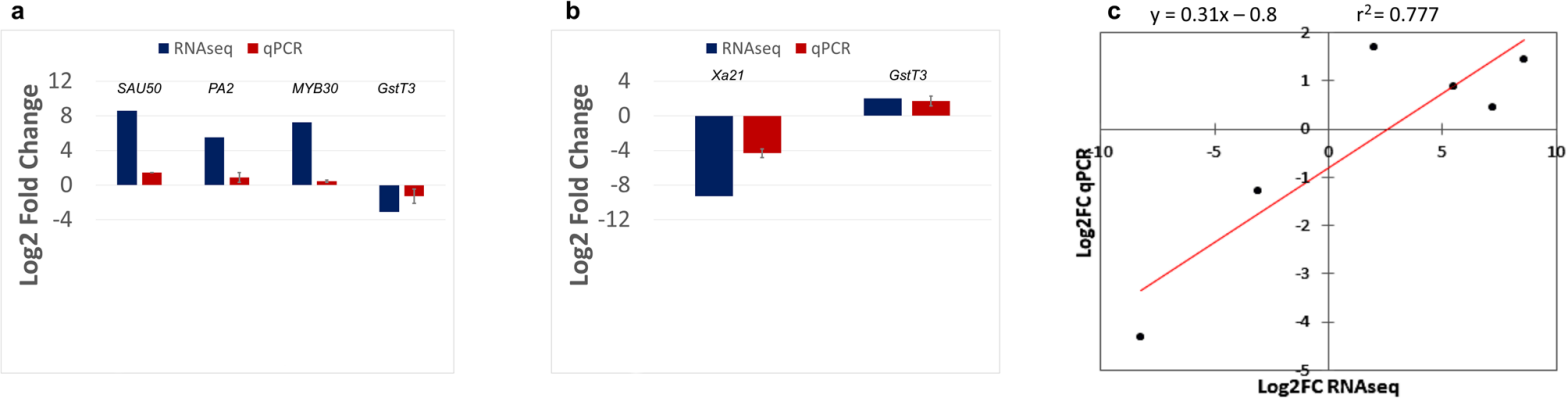
Validation of RNASeq data with qPCR. (**a**) and (**b**) Expression of five randomly selected genes was examined by qPCR analysis. For each gene, fold changes were calculated by ΔΔCt method and log2Fold change were compared between qPCR and RNAseq. (**c**) Correlation between RNAseq and qPCR data based on log2fold change of the five selected genes: y = 0.31x – 0.8.

Five genes differentially expressed under or not treatment of MeJA of SosatC88 and Souna3 were selected based on log2 fold change and adjusted p-value parameters (FDR < 0.05) and their expression was validated by qPCR. These DEGs encode for: a small Auxin Up-Regulated RNA 50 (SAUR50), a peroxidase A2 (PA2), a myeloblastosis 30 (MYB30), a receptor kinase-like protein Xa21 and a glutathione S transferase T3 (GstT3). These DEGs included plant hormone signal transduction related genes, stress-responsive genes, peroxidase activity-related genes. Differential expression of transcripts was observed in SosatC88 and Souna3 between non-treated and MeJA-treated plants in concordance with the RNAseq data (Fig. 8a,b). The results also validated the contra-regulation revealed in RNAseq of *PgGstT3* by MeJA treatment in SosatC88 and Souna3. A Pearson correlation was performed to test whether the qPCR results correlated with the RNAseq results. The ratio of expression levels obtained between non-treated and treated plants by qPCR was compared to the ratio of expression measured by RNA-Seq. A significant correlation (r^2^ = 0.777, *p* = 0.02) was observed between qPCR and RNA-Seq data that validate the differential expression of genes (Fig. 8c).

## Discussion

Water deficit is considered one of the most threatening abiotic stresses in agriculture in the era of climate change^31^. Water deficit resulting from these drought periods affects different aspects of plant growth and development, leading in some cases to a considerable crop production loss. These negative impacts can be alleviated by a wide range of plant hormonal treatments. MeJA, which is known to be involved in stress resistance mechanisms in plants, may have a key role to play in providing useful solutions. MeJA significantly reduced growth and leaf expansion in soybeans (*Glycine max*), hampered seedling diameter in scots pine (*Pinus sylvestris*)^32–34^, and inhibited both root and shoot growth in rice (*Oryva sativa*)^35^. The treatment of MeJA 200 µM at the early stage of pearl millet development did not induce significant visible morphological change between genotypes. However, the MeJA-treated plants retained more vigor than the non-treated plants. Similar results were observed in rice (*Oryza sativa* L.) which the drought-sensitive genotype raised with primed seeds with 100 µM of MeJA retained its morphology better than the tolerant genotype under drought condition^36^. In wheat (*Triticum aestivum* L.), 0.25 µM of MeJA delayed senescence under drought condition^37^. In pearl millet, key characteristics of drought resistance are related to root morphology as well as an efficient photosynthetic machinery^30^. Our results revealed a reduction in chlorophyll content that suggests a loss of photosynthetic pigments or activity. These are in accordance with previous studies^33,38,39^ that observed a decline in photosynthesis of soybean (*Glycine max*), barley (*Hordeum vulgare*) leaves and *Arabidopsis thaliana*. The loss of photosynthetic pigments would decrease the amount of energy absorbed by the photosynthetic system, thus reducing the energy required for metabolism, which consequently impacts plant growth and development. The loss of chlorophyll content could also be attributed to leaf senescence^40^. Furthermore, it has been shown that jasmonates attenuate the influence of drought by favoring the closure of stomata^26,41^. Our findings suggest that treatment of MeJA could cause the stomatal closure, leading to the inhibition of CO_2_ absorption and the reduction of photosynthetic activity^11,42,43^.

Despite non-significant morphological changes between genotypes, MeJA treatment was effective in modulating endogenous stress-related genes expression. It is known that gene encoding Lox2 is highly induced by jasmonates (JA and MeJA) or in response to biotic stress^44^ and NCED is the key enzyme in the ABA biosynthetic pathway in plants^45,46^. The overexpression of pearl millet Lox2 (*PgLox2*) is induced by the jasmonates produced by the plants in response and in response to MeJA treatment. The expression profile of pearl millet NCED (*PgNCED*) in MeJA-treated and non-treated plants suggests that water deficiency and MeJA treatment induced an increase in ABA biosynthesis, which is most important with external MeJA input. These results suggest that the decrease in plant growth is caused by increased ABA and MeJA accumulation.

To gain insights in the molecular basis of pearl millet responses to MeJA treatment, a transcriptome analysis was carried out at the early stage of plant development. Transcriptomic data were generated to identify the molecular basis at the genome-wide level underlying the response of pearl millet to MeJA treatment at the early developmental stage. Under non-treated conditions, distribution of DEGs in drought-sensitive SosatC88 and drought-tolerant Souna3 in extracellular region term, pathogenesis term and transferase activity, transferring acyl group term according to GO analysis suggested that water-deficiency involves genes related to these terms. In contrast, when MeJA was applied there was no significant difference in the transcriptomic response between SosatC88 and Souna3. This suggests that MeJA stimulates in SosatC88 pathways and biological processes to better adapt to water deficiency as in Souna3. In addition, 12 genes were found to be differentially expressed in the opposite manner depending on whether we are in MeJA-treated or non-treated conditions. Among these genes, two are up-regulated by the MeJA treatment. These two genes are *Pgl_GLEAN_10004431* encoding for an unknown protein and *Pgl_GLEAN_10018402* encoding for an uncharacterized protein family UPF0136 transmembrane. The additional ten genes were down-regulated by the MeJA treatment. They are mostly *Tyrosine-protein kinase* (*Pgl_GLEAN_10005256*), *Multicopper oxidase, type 2* (*Pgl_GLEAN_10001778*), *Peptidase* (*Pgl_GLEAN_10016112*), *Phospholipid/glycerol acyltransferase* (*Pgl_GLEAN_10012582*), *Glycoside hydrolase* (*Pgl_GLEAN_10004142*) and uncharacterized genes. About the uncharacterized genes, they could likely represent key regulators of the variation in flowering time or drought tolerance among different genotypes. Further investigation is required for understanding the role in pearl millet adaptation to stress.

Data reported here indicate in one hand that the MeJA triggers higher transcriptional responses in SosatC88 to better adapt and cope with water deficiency in opposite to Souna3. The striking difference in DEGs between SosatC88 and Souna3 provides information on the amplitude of the effect of the MeJA treatment on the transcriptome of SosatC88. Consistent with data reported here, other transcriptome studies report a higher number of DEGs under water stress-sensitive variety in wheat (*Triticum aestivum*)^47^ and sorghum (*Sorghum bicolor*)^48^. MeJA induced in both genotype enrichment of GO terms related to terpene synthetase activity and lyase activity. One way to protect against oxidative stress is the biosynthesis of volatile terpenoids, which is thought to quench ROS^49^. Lyases are known to play a role in antioxidant activity in cases of biotic or abiotic stress^50^. We theorize that these groups of genes probably constitute the characteristic basis for the adaptation to water deficit conferred by the treatment of MeJA in both genotypes SosatC88 and Souna3. On the other hand, the results of the GO enrichment analysis of up-regulated gene groups provide evidence of striking antioxidant enzyme activities. Several important functional gene groups related to water deficiency stress including oxidation–reduction process, heme binding, electron transfer activity, response to oxidative stress, membrane, peroxidase activity, among others, are significantly enriched in SosatC88 treated with MeJA. Peroxidases are involved in the defense against abiotic stress through their role in the scavenging of ROS^51^. They are also involved in cell wall formation, confer cell wall rigidity and reducing leaf expansion^32^. It has also been shown in several studies that MeJA treatment increases peroxidase activity^32,37,39^. A cross-species meta-analysis of progressive drought stress identifies the heme-binding family protein as key drought adaptive genes conserved^52^. Our results show that MeJA improves SosatC88 adaptation, drought-sensitive genotype, to water deficiency by increasing and diversifying its antioxidant response mechanisms to cope under these unfavorable conditions.

On the other hand, proteins such as peroxidase superfamily protein, thioredoxin-dependent peroxidase, prohibitin 3 and a subunit of the NAD(P)H complex located in the chloroplast thylakoid lumen (Pgl_GLEAN_10037666) identified with high level in pearl millet leaves under drought stress ^29^ have their genes expression up-regulated under drought stress conditions. These groups of proteins were also up-regulated in the leaves under drought in bean (*Phaseolus vulgaris* L.^53^, in wheat (*Triticum aestivum* L.)^54^ and in maize (*Zea mays)*^55^. Furthermore, ribulose bisphosphate carboxylase small chain (Pgl_GLEAN_10020566), a protein identified as associated with a degradation of chlorophyll^30^. Together, they are associated with the processes that underlie the senescence or the stay-green phenotype and overexpressed in SosatC88 by MeJA. This shows the importance of these proteins as they have passed post-transcriptional regulation to play a major role in the response to drought.

## Conclusion

A successful treatment of exogenous MeJA in two genotypes with different response to drought did not alter plant morphology, but modulated gene expression. RNAseq analysis revealed a transcriptional process that mobilizes a large number of DEGs related to cell wall protection and detoxification, implying different mechanisms involved in response to drought at the early stage of the pearl millet developmental cycle. Five genes differentially expressed are genuine candidates as molecular markers in functional genomics or in the breeding of pearl millet. Overall, data give an indication that, in pearl millet, exogenous MeJA treatment may play a dual role by simulating abiotic stress defenses to protect plants under water deficit and contribute to crop production in drought-prone environments.

## Materials and methods

### Plant material and growth Conditions

Seeds of two pearl millet open-pollinated varieties, SosatC88 and Souna3, were used. SosatC88 was developed by recombining 19 S1 progeny selected at Cinzana (Mali) in 1988 from a cross between the local Souna and Sanio genotypes. It was considered a water deficit-sensitive. Souna3 is a synthetic variety resulting from the recombination of eight lines from the PC 28 and PC 32 populations (106–7, 108–4, 113–3, 115–4, 134–5, 142–4, 143–4, 148–3). Souna3 is a drought tolerant genotype which reaches maturity between 85–95 days and can be grown in areas with moderate rainfall ranging between 400 and 750 mm. All plant material used in this study are provided by the ISRA genebank and their uses comply with relevant institutional, national, and international guidelines and legislation.

The experiment was performed in the shadehouse of the Centre d'Etude Régional pour l'Amélioration de l'Adaptation à la Sécheresse (CERAAS) in Thiès (Sénégal; 14°45′57.17″N, 16°53′31″W) from March to April 2018 with a photoperiod of 13 h of light/day with a light intensity of 1158 µmol lux, a vapor pressure deficit (Vpd) of 2.3 Kpascal, an average air temperature of 35.3 ºC day and 23.5 ºC night.

Both genotypes were sown in 10 L plastic buckets containing a non-autoclaved sandy soil with low levels of clay and silt (12%) and organic matter (0.4%) from Bambey (Senegal)^7^. In each bucket, 4 plants of the same variety were grown. These buckets were completely randomized with 6 replicates per treatment.

The plants were watered three times a week by pouring an equivalent volume of water on each soil bucket until 17 days after sowing (DAS). Then, water was withdrawn and plant leaves were sprayed with a solution containing 200 μM MeJA (Methyl jasmonate 95%, CAS Number: 39924–52-2 (Methyl 3-oxo-2-(2-pentenyl) cyclopentaneacetate from Sigma-Aldrich) dissolved in 0.1% tween 20 and with 0.1% tween 20 for the MeJA-treated and the non-treated, respectively, every day for 10 days^24^. Three days after the last treatment, the aerial parts (shoot and leaf) of the MeJA-treated and non-treated plants were collected at late seedling growth stage (i.e. phase 17 according to the BBCH scale^56^, https://en.wikipedia.org/wiki/Cereal_growth_staging_scales), immediately frozen in liquid nitrogen and stored at – 80 °C for further RNA extraction.

### Development and Chlorophyll density parameters measurement

Leaf number and plant height were assessed at T0, T1 and T2. T0 corresponds to the first day just before treatment (both non-treated and MeJA-treated plants), T1 and T2 correspond to 5 and 10 days after treatment, respectively. The chlorophyll density level was measured at T1 and T2 using a SPAD 502 PLUS CHLOROPHYLL-METER^57^. Four plants from the same pot were used for measurement of leaves number, plant height and chlorophyll density level, which resulted in six biological replicates for each genotype in each sampling date. A significant difference in mean between the samples as obtained by Student’s t-test at P < 0.05.

### RNA extraction

Leaves from four plants of each genotype were pooled in one sample. Sampling was performed on three biological replicates (MeJA-treated and non-treated plants)^58^. The samples were ground in liquid nitrogen and then, 200 mg of powder was used for total RNA isolation with TRI Reagent (Sigma-Aldrich) according to the instructions of the manufacturer. RNA was then DNase treated (Qiagen, Germany) and purified using Qiagen RNeasy Minikit. The quality of the RNA was evaluated on 1.2% agarose gel (Supplemental figures, Fig. S2) and electrophoresis was carried out at 100 Volts for 30 min and the quantification was performed using a Nano Drop Lite and the Agilent 2100 bioanalyzer.

### Gene markers expression level assay for MeJA treatment validation

To validate the results observed at the end of the MeJA treatment, we identified genes that are involved in response to plant biotic and abiotic stresses^24^. Among these, we used *Lipoxygenase 2* (*Lox2*) involved in the biosynthesis of MeJA and *9-cis-epoxycarotenoid* (*NCED*) involved in abscisic acid (ABA) biosynthesis. Both are associated in plant response to abiotic/biotic stresses. *Lox2* encodes the enzyme that catalyzes an important step in jasmonic acid (JA) biosynthesis from linolenic acid derived from the membrane^59,60^ while NCED is the key enzyme in ABA biosynthesis involved in plant response to water deficit^46^.

Primer pairs for *PgLox2* and *PgNCED* were designed using Primer3Plus online tool^61^ according to the target gene-specific sequence design, the option of qPCR setting Primer3Plus online tool platform was used. The primer sequences of transcripts are listed in Supplemental file, Table S2.

### qPCR assays

Five hundred (500) ng of the total RNA was reverse transcribed using the Goscript Reverse Transcriptase system (Promega) according to the manufacturer’s instructions. The qPCR reaction was performed in a total volume of 10 µl containing 5 µl of GoTaq® qPCR Master Mix (2X), 0.25 μM of each primer, 1 μl (100 ng) of diluted cDNA and free nuclease water and then, loaded in a StepOnePlus™ Real-Time PCR Systems. The program used for qPCR contains three steps: the first step is the initial activation at 95 °C for 2 min; the next step is the PCR amplification performed up to 40 cycles with 95 °C for 15 s, 58 °C for 60 s and the third step is a melt curve analysis ramping from 58 °C to 95 °C.

The relative expression levels of transcripts under the different conditions were normalized based on the expression of an endogenous control gene, *ubiquitin 5 (UBQ5)*^62^ using the 2^−∆∆Ct^ method^63^. The qbase + software Version: 3.2^64^ was used for analysis.

### RNAseq

#### Library construction and sequencing

For library construction, RNAs were purified on RNAClean XP beads and then analyzed by capillary electrophoresis (Fragment Analyser). Highly pure mRNA was isolated from the total RNA using oligo (dT) beads. The Illumina TruSeq RNA Library Prep Kit v2 was used to synthesize the second strand cDNAs library. Sequencing was performed on an Illumina HiSeq 2500 using the Sequence By Synthesis (SBS) technique using the TruSeq Rapid SBS kit (Illumina). Sequencing results were obtained as single-end reads (50 bp each) in the FASTQ format.

#### Differential gene expression analysis

Clean reads were aligned to the reference genome^5^ and counted with Bioconductor R package Rsubrea^65^. The read counts were used to performed the Differential Expression Genes analysis with edgeR package of Bioconductor^66^. Pairwise comparisons were performed between four datasets: non-treated SosatC88 vs non-treated Souna3, MeJA-treated SosatC88 vs MeJA-treated Souna3, MeJA-treated SosatC88 vs non-treated SosatC88, and MeJA-treated Souna3 vs non-treated Souna3. False Discovery Rate (FDR) < 0.05 and log|Fold change|> 2 were considered as the conditions to state the genes as differentially expressed.

#### Functional annotation of transcripts and genes enrichment analysis

The sequence similarity search was carried out against the National Center for Biotechnology Information (NCBI) non-redundant (nr) protein database and the Swiss-Prot protein database using the BLASTx algorithm from Blast2GO^67^ specifying E-values < 10^–5^. Gene Ontology (GO) categorization was done with Blast2GO. From the annotated GO file, we classified and plotted the DEGs according to the official classification (Molecular Function, Biological Process and Cellular Component), using WEGO 2.0^68,69^ and functional enrichment analysis was also performed using Goseq^70^, a bioconductor R package implemented in galaxy^70^ using Wallenius method.

DEGs were then mapped on the biological pathways of the web-based Kyoto Encyclopedia of Genes and Genomes (KEGG) web-based annotation server (KASS) by running Blastx against KEGG GENES (Kyoto Encyclopedia) https://www.genome.jp/kegg/kaas/. We completed the KEGG annotation using the KEGG result files of millet genome information uploaded to GIGAdb^71^. The result contains KO assignments (KEGG Orthology) and KEGG pathways were automatically generated. KEGG enrichment analysis was also performed based on the same criteria described above for GO.

## Supplementary Information

Supplementary Information 1.

Supplementary Information 2.

## Data Availability

All data provided in the article and Supplementary files are available from the corresponding author on reasonable request.
