# Supplementary material for: Transcriptomic analysis of methyl jasmonate treatment reveals gene networks involved in drought tolerance in pearl millet
Source: Sci Rep. 2022 Mar 25;12:5158. doi: 10.1038/s41598-022-09152-6 (PMC8956577; doi:10.1038/s41598-022-09152-6)

**Transcriptomic analysis of methyl jasmonate treatment reveals gene networks involved in drought tolerance in pearl millet**

Adama Ndiaye ^1,2,3^, Amadou Oury Diallo ^1,3^, Ndèye Coura Fall ^1^, Rose Diambogne Diouf ^1^, Diaga Diouf ^2,3^, Ndjido Ardo Kane ^1,3,*^

# Supplementary figures


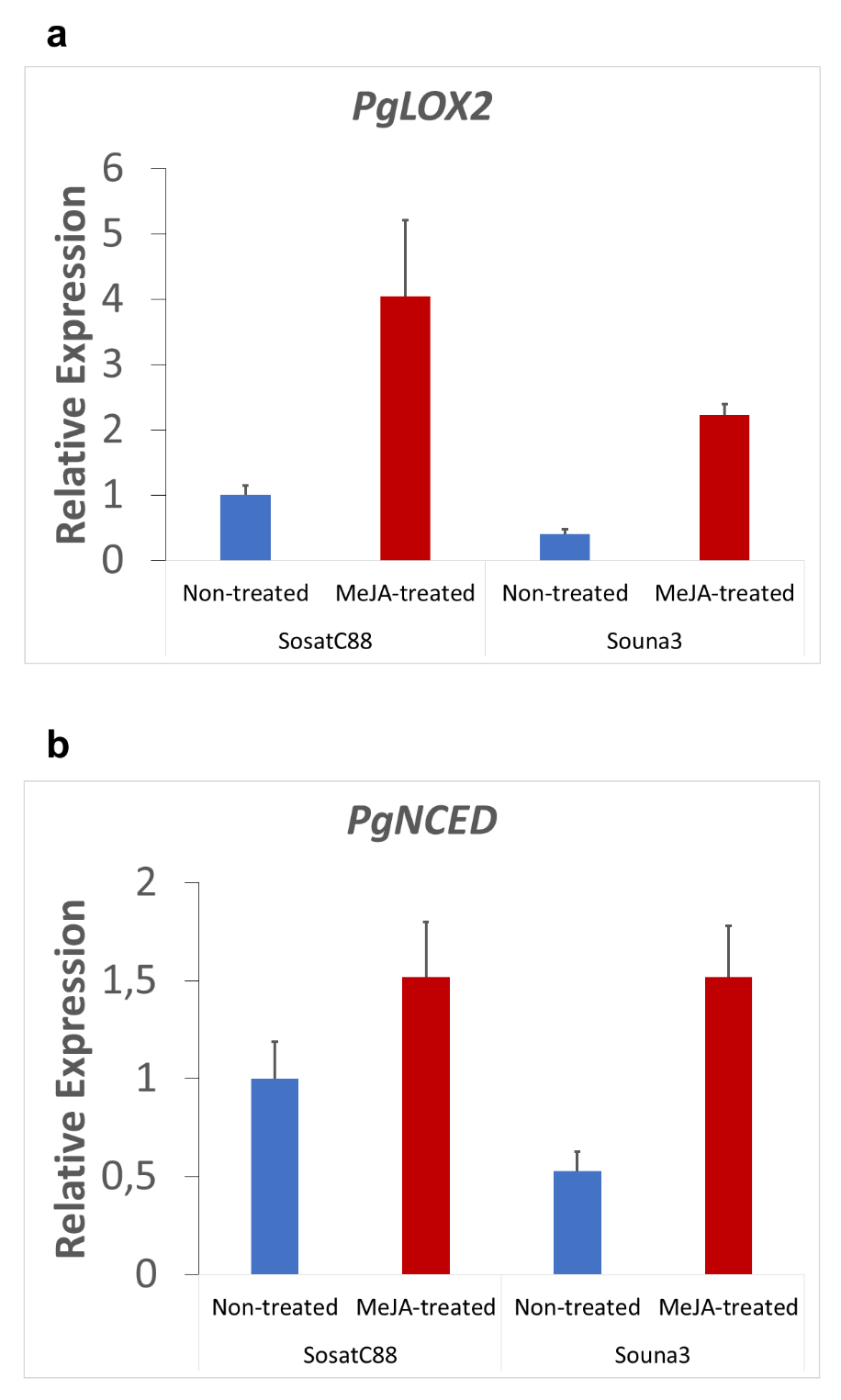


Figure S 1: Effect of exogene methyl jasmonate on expression level of PgNCED and PgLox2 in SosatC88 and Souna3

Figure S 2: 1.2 % agarose gel electrophoresis showing RNA extraction by TRI Reagent (Sigma-Aldrich) method and purified using Qiagen RNeasy Minikit of P. glaucum treated or not by MeJA.


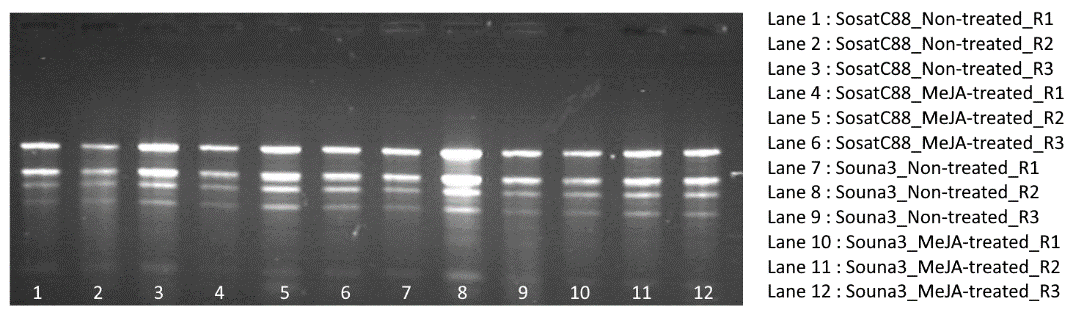

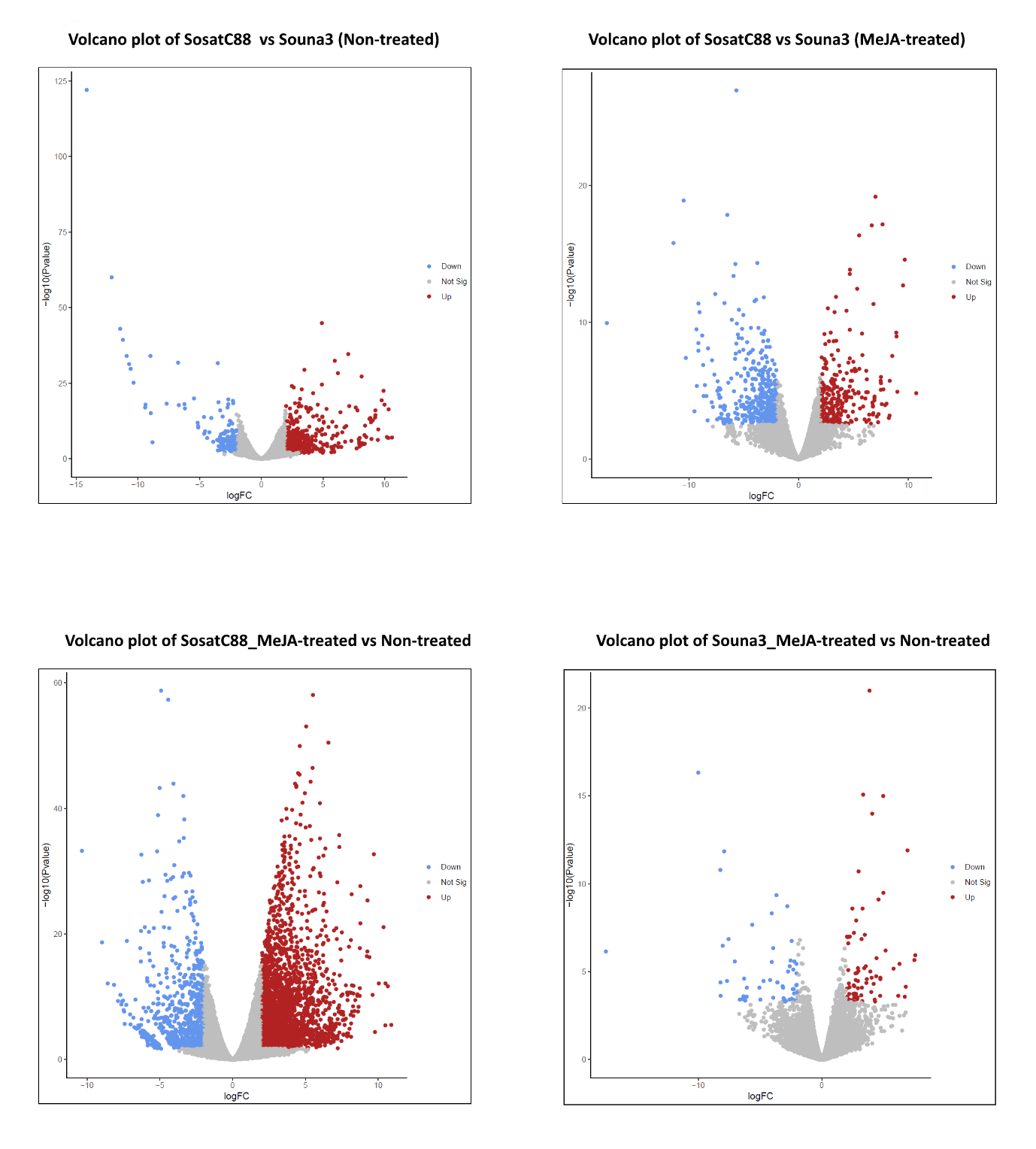


Figure S 3 : Volcano plots of DEGs in different datasets

Figure S 4: a) Venn diagram of number of differentially expressed genes shared and distinct to ‟SosatC88 vs Souna3 (non-treated)” and ‟SosatC88 vs Souna3 (MeJA-treated)”; b) Venn diagram of number of differentially expressed genes shared and distinct to ‟SosatC88_MeJA- treated vs non-treated” and ‟Souna3_MeJA- treated vs non-treated”. Oliveros, J.C. (2007-2015) https://bioinfogp.cnb.csic.es/tools/venny/index.html


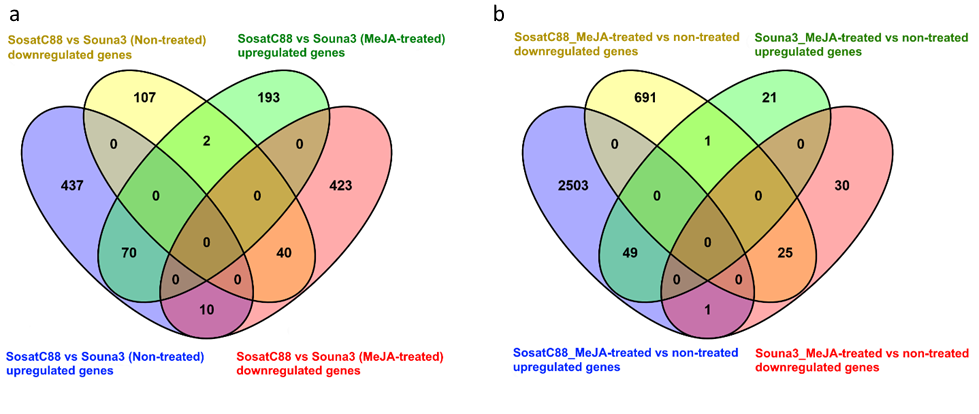

Supplement: Supplementary file 1 — Supplementary Information 1. [file 41598_2022_9152_MOESM1_ESM.docx]
